# Effect of electrical stimulation therapy of the lower esophageal sphincter in GERD patients with ineffective esophageal motility

**DOI:** 10.1007/s00464-020-08104-3

**Published:** 2020-10-30

**Authors:** Matthias Paireder, Ivan Kristo, Reza Asari, Gerd Jomrich, Johannes Steindl, Erwin Rieder, Sebastian F. Schoppmann

**Affiliations:** Department of Surgery, Upper-GI-Service, Comprehensive Cancer Center GET-Unit, Medical University of Vienna, Vienna, Austria

**Keywords:** Electrical stimulation therapy, Gastro esophageal reflux disease, Ineffective esophageal motility, Lower esophageal sphincter

## Abstract

**Background:**

Electrical stimulation therapy (EST) of the lower esophageal sphincter (LES) is a novel technique in antireflux surgery. Due to the minimal alteration at the LES during surgery, LES-EST is meant to be ideal for patients with gastroesophageal reflux disease (GERD) and ineffective esophageal motility (IEM). The aim of this prospective trial (NCT03476265) is to evaluate health-related quality of life and esophageal acid exposure after LES-EST in patients with GERD and IEM.

**Methods:**

This is a prospective non-randomized open-label study. Patients with GERD and IEM undergoing LES-EST were included. Follow-up (FUP) at 12 months after surgery included health-related quality of life (HRQL) assessment with standardized questionnaires (GERD-HRQL) and esophageal functional testing.

**Results:**

According to the study protocol, 17 patients fulfilled eligibility criteria. HRQL score for heartburn and regurgitation improved from 21 (interquartile range (IQR) 15–27) to 7.5 (1.25–19), *p* = 0.001 and from 17 (11–23.5) to 4 (0–12), *p* = 0.003, respectively. There was neither significant improvement of esophageal acid exposure nor reduction of number of reflux events in pH impedance measurement. Distal contractile integral improved from 64 (11.5–301) to 115 (IQR 10–363) mmHg s cm, *p* = 0.249. None of the patients showed any sign of dysphagia after LES-EST. One patient needed re-do surgery and re-implantation of the LES-EST due to breaking of the lead after one year.

**Conclusion:**

Although patient satisfaction improved significantly after surgery, this study fails to demonstrate normalization or significant improvement of acid exposure in the distal esophagus after LES-EST.

Antireflux surgery is recommended, if lifestyle modification and medical treatment (antacids, proton pump inhibitors (PPI), and histamine 2 (H2−) receptor antagonists) for gastroesophageal reflux disease (GERD) fail [1, 2]. Prior to surgery, manometry and pH metry are crucial tools to assess the presence of GERD and exclude esophageal motility disorders like outflow obstruction, achalasia, or other contractility disorders such as hypercontractile esophagus or absent contractility that may affect outcomes [3, 4]. Interestingly, the evolution of high-resolution manometry re-defined motility disorders like ineffective esophageal motility (IEM) [5]. IEM is known to be associated with advanced GERD highlighted by a high reflux burden during objective testing, whereas its surgical management in context of GERD is still a matter of an ongoing debate [6, 7]. Postoperative dysphagia is a bothersome adverse event that is observed in up to 70% of the patients with severe motility disorders, who undergo laparoscopic fundoplication [8]. Although this may resolve after some period of time or even after interventions, this is still a very troublesome period of time for the patient.

A novel approach of surgical antireflux treatment was the concept of an electrical stimulation therapy (EST) of the lower esophageal sphincter (LES) [9]. It was shown that LES-EST significantly increases the LES pressure without affecting esophageal peristalsis or LES relaxation [10]. Furthermore, the two-year results of a prospective open-label trial were rather promising revealing a significant improvement of symptomatic GERD and esophageal acid exposure [11]. The idea to improve the competence of the LES without changing anatomical structures is an interesting novel strategy for patients with GERD and IEM.

Therefore, this prospective study was designed to evaluate the impact of LES-EST in patients with GERD and IEM. In a preliminary assessment one month after surgery, we could show that there was no impact on postoperative dysphagia and good early symptom control [12].

This is the one-year report of patients with GERD and IEM treated with LES-EST.

## Material and methods

### Study protocol

This is a prospective, non-randomized, open-label study. The study protocol including inclusion and exclusion criteria was described in detail elsewhere [12]. In brief, patients who fulfilled the criteria of IEM according to the Chicago classification v3.0 were prospectively screened for eligibility [3].

Preoperative workup included upper GI endoscopy as well as esophageal functional testing (EFT) including high-resolution impedance manometry (InSIGHT Ultima®, Sandhill Scientific Inc., USA) and 24 h impedance/pH reflux monitoring (ZepHr®, Sandhill Scientific Inc., USA). Patients were off PPI 15 days prior to testing.

Follow-up took place after 1, 6, and 12 months after surgery and included physical examination, interrogation of the device, and health-related quality of life (HRQL) assessment with standardized questionnaires (GERD-HRQL for heartburn and regurgitation) [13]. Dysphagia was graded from 0 to 4, according to the standardized classification used by Mellow and Pinkas [14]. Esophageal functional testing was repeated during follow-up and presented in this analysis. In patients under PPI treatment, the medication was stopped 15 days prior to EFT.

The ethical committee (EK1217/2017) of the Medical University of Vienna approved the study. All patients gave their written informed consent. This prospective study is registered at clinicaltrials.gov (NCT03476265).

### Implantation technique

All patients included in this analysis received laparoscopic implantation of an EndoStim Generation II device (Generation-II-LES-Stimulator Modell 1006). If a hiatal hernia existed, posterior hiatal hernia repair was performed. Two stimulation electrodes were placed under endoscopic control at the anterior side of the gastroesophageal junction approximately 1.5 cm apart and were fixed with 3/0 multifilament, non-absorbable thread, which is applied at least at one side of each silicone butterfly. Correct position of the electrodes and function of the stimulation device was verified using the external programmer. A contrast swallow with Diatrizoate was performed at day one after surgery as well as an abdomen X-ray for documentation of the position of the lead and electrodes. Patients were encouraged to take in soft diet for 4 days [12]. Further medical and technical details of the device and the programming unit were extensively described by Rodriguez 2015 [11].

### Statistical analysis

SPSS (Version21.0, SPSS Inc., Chicago, IL, USA) was used for statistical analysis.

All variables were depicted as median and interquartile range (IQR) or 95% confidence intervals (CIs) or mean with standard deviation (SD). GERD health-related quality of life (HRQL) score and esophageal acid exposure before and after treatment were compared with paired Wilcoxon test. *P* values < 0.05 were considered significant. Graphing was performed with GraphPad Prism (Version 7.0c, GraphPad Software, Inc., La Jolla, CA, USA).

## Results

### Patient characteristics

Seventeen patients (male = 11, 64.7%) fulfilled the eligibility criteria and underwent implantation of LES-EST with a mean age of 48.9 (SD12.6) years and a mean body mass index of 25 (SD 4.8). Based on BMI, 35.3% of patients were classified as overweight (BMI ≥ 25—< 30) or obese (BMI ≥ 30). Prior to surgery, 11 (64.7%) patients were taking PPI on a regular basis. Twelve patients (70.65%) presented a hiatal hernia. The size of the hernia did not exceed the Hill grade III and were all classified in the preoperative endoscopy as maximum medium sized. All patients had a bothersome history of symptomatic GERD highlighted by a median baseline GERD-HRQL score of 43 (IQR 22–47). Esophageal functional testing revealed a median % time of pH < 4 of 8.9 (IQR 4–21.6) and a median DCI of 64 mmHg s cm, IQR 11.5–301). Further baseline patient characteristics are outlined in Table 1. Details regarding EFT are displayed in Table 2.

**Table 1:** Patient characteristics

Characteristic	Baseline	12-month follow-up	*P*^*§*^
Age, years*	48.9 (12.6)°		
Body mass index (BMI)*	25.0 (4.8)°		
Gender			
Male	11 (64.7)		
Female	6 (35.3)		
BMI class			
Normal (< 25)	11 (64.7)		
Overweight (≥ 25 and < 30)	4 (23.5)		
Obese (≥ 30)	2 (11.8)		
Patients using daily PPI	11 (64.7)	4 (23.5)	
Typical GERD symptoms	11 (64.7)		
Atypical GERD symptoms	9 (52.9)		
GERD-HRQL score			
Heartburn (IQR)	21 (15–27)*	7.5 (1.25–19)*	*0.001*
Regurgitation (IQR)	17 (11–23.5)*	4 (0–12)*	*0.003*
Total (IQR)	43 (22–47)*	12 (3.5–20.8)*	*0.003*

Values in parentheses are percentages unless indicated otherwise

*PPI* proton pump inhibitors, *GERD* gastroesophageal reflux disease, *HRQL* health-related quality of life, *§* Paired Wilcoxon test

°Values are mean (standard deviation, SD)

*Values are median (interquartile range, IQR)

**Table 2:** Esophageal functional testing – 12-month follow-up

Characteristic	n	Baseline	n	12-month follow-up	*P°*
Esophageal functional testing					
Total % pH time < 4	17	8.9 (4–21.6)	14	10.4 (5.6–16.7)	*0.551*
Upright % pH time < 4	17	7.8 (1.5–20.3)	14	6.3 (2.8–11.2)	*0.109*
Supine % pH time < 4	17	14.4 (1.7–20.7)	14	14.7 (5.0–19.4)	*0.802*
Nr of reflux events	17	81 (52.3–100.8)	10	73 (39.3–114.5)	*0.386*
LES resting pressure (mm HG)	17	15 (12.7–23.4)	14	14.6 (6.3–17.2)	*0.036*
DCI mmHg s cm	17	64 (11.5–301)	14	115 (10.0–363.0)	*0.249*

Values are median (interquartile range, IQR)

*LES* lower esophageal sphincter, *DCI* distal contractile integral

***°***Paired Wilcoxon test

### Implantation and LES-EST treatment parameters

All procedures were performed minimally invasive by the same surgical team. Median operating time was 45 min (IQR 34–61). In twelve patients (70.65%), a posterior hiatal repair was done. The absence of perforation caused by the electrodes was documented during intraoperative upper gastrointestinal endoscopy. Stimulation parameters were set to 16 sessions for 20 min with 5 mAmp in all patients. Median impedance baseline was 328 (IQR 301.75–366) Ohm. There were no adverse events during surgery.

### Twelve-month follow-up

**Fig. 1:**
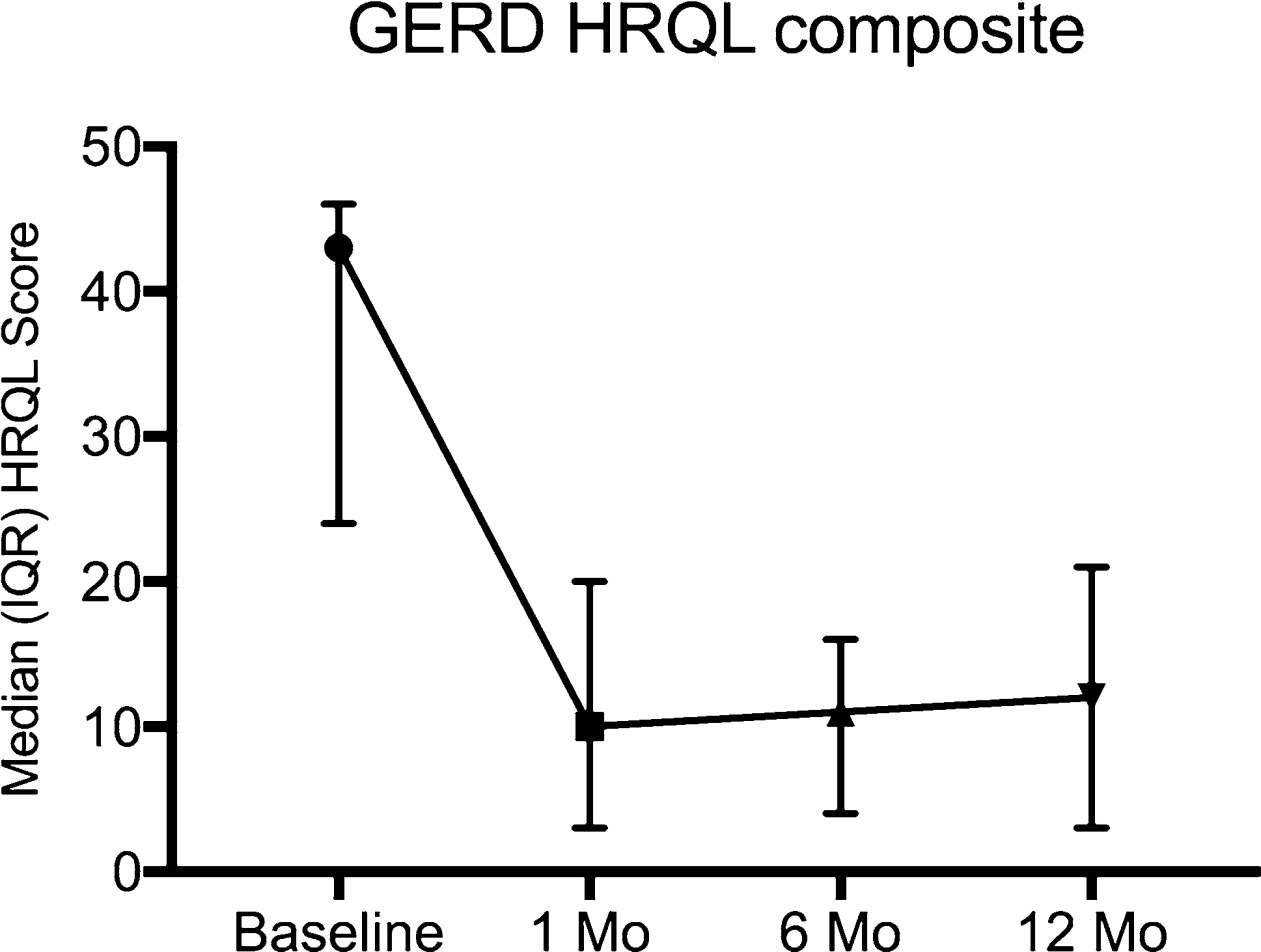
Change in median (interquartile range—IQR) GERD health-related quality of life (HRQL) composite score. *P* = 0.003 versus baseline and 12-month follow-up

**Fig. 2:**
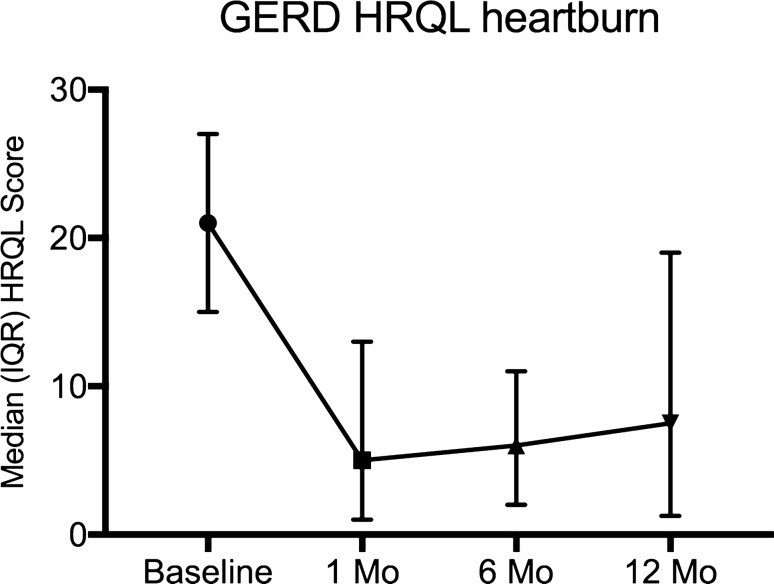
Change in median (interquartile range—IQR) GERD health-related quality of life (HRQL) heartburn score. *P* = 0.001 versus baseline and 12-month follow-up

**Fig. 3:**
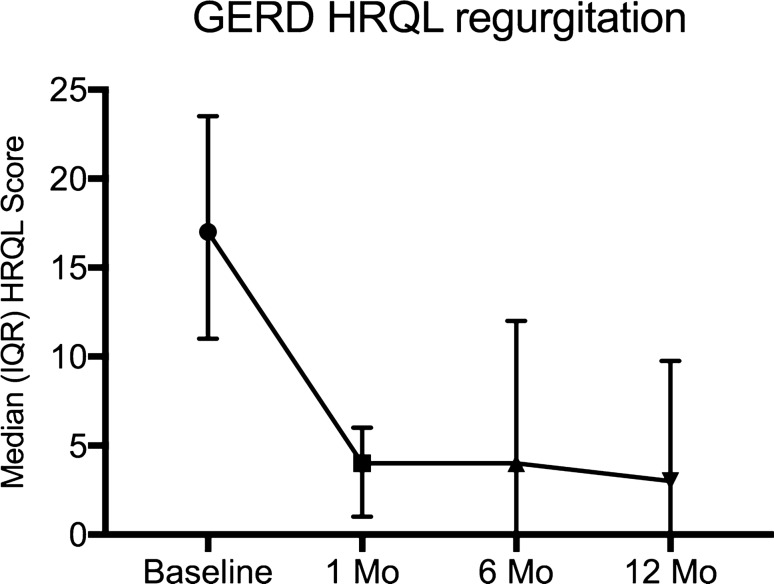
Change in median (interquartile range—IQR) GERD health-related quality of life (HRQL) regurgitation score. *P* = 0.003 versus baseline and 12-month follow-up

All patients fulfilled clinical follow-up and interrogation of the device one year after surgery. HRQL score for heartburn and regurgitation improved to 7.5 (IQR 1.25–19), *p* = 0.001 and to 4 (IQR 0–12) *p* = 0.003, respectively. Composite HRQL score 12 months after surgery was 12 (3.5–20.8), *p* = 0.003; Figs. 1, 2, and 3. Four patients (23.5%) were taking PPIs at least intermittently. 12 months after surgery impedance rose to 514.6 (IQR 412 – 583.5).

**Fig. 4:**
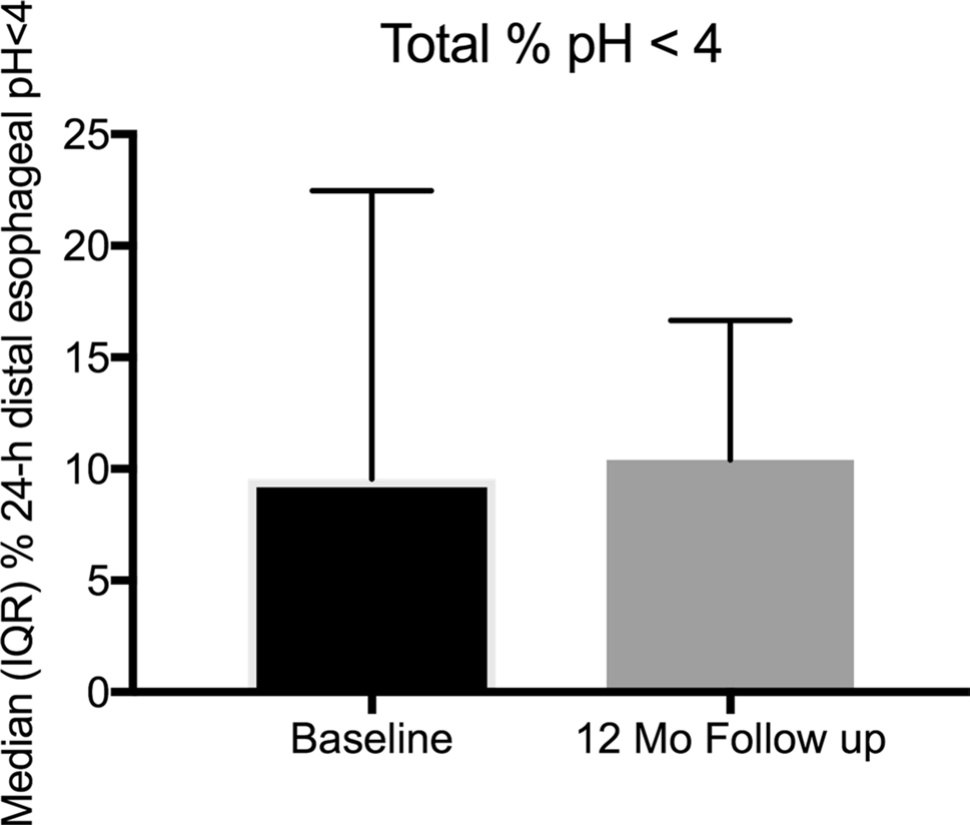
Median (interquartile range – IQR) % of time 24-h distal esophageal pH at baseline and 12-month follow-up

**Fig. 5:**
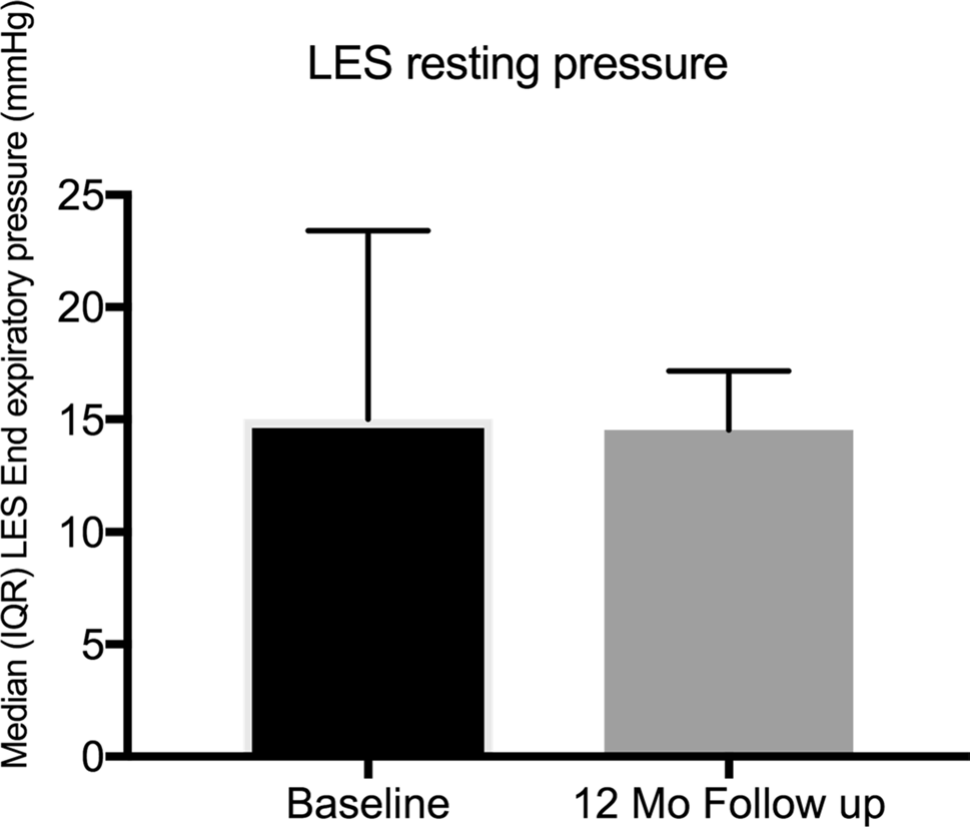
Median (interquartile range – IQR) lower esophageal sphincter (LES) resting pressure at baseline and 12-month follow-up

**Fig. 6:**
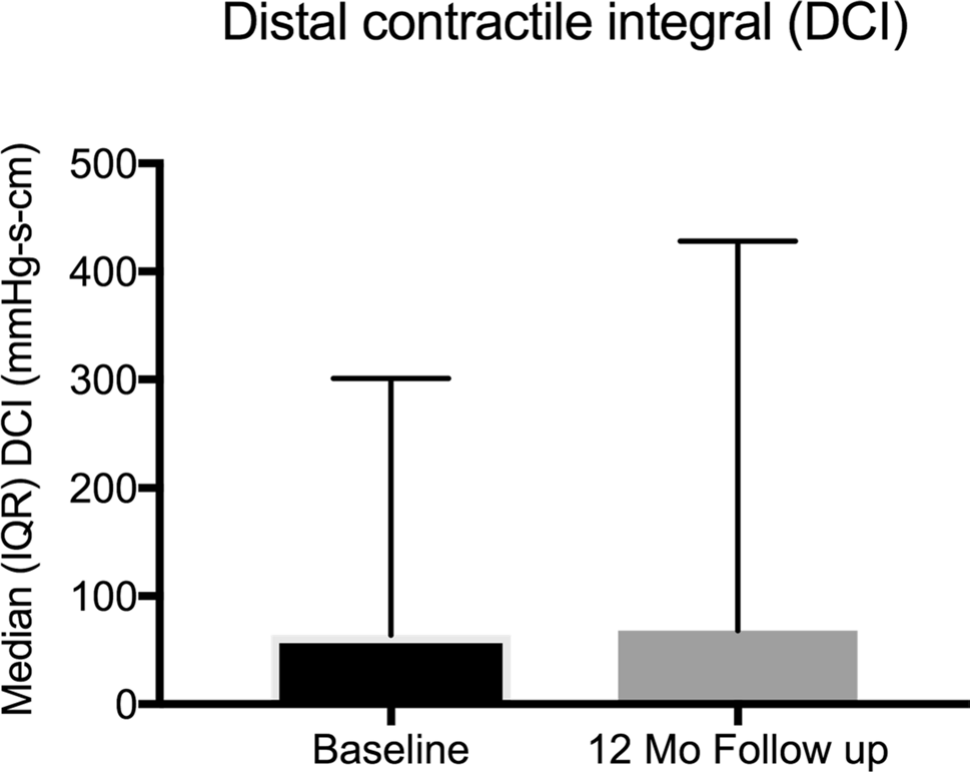
Median (interquartile range – IQR) distal contractile integral (DCI) at baseline and 12-month follow-up

Follow-up esophageal functional testing was performed in 14 (82.4%) patients. There was neither significant improvement of acid exposure nor reduction of number of reflux events in pH impedance measurement. DCI improved to 115 (IQR 10–363) mmHg s cm (*p* = 0.249), registered during manometry. Three (17.6%) patients returned to regular esophageal motility. There were no patients that experienced dysphagia. Further details of the EFT at follow-up are displayed in Table 2 and Figs. 4, 5, and 6. We observed no differences in outcome parameters between patients with or without cruroplasty.

In one patient, 6 months after surgery, the interrogation of the device showed the impedance out of reach. This patient underwent re-laparoscopy and exchange of the stimulation electrodes and pulse generator. The technical assessment of the defective device showed a leakage of the insulation sleeve of the cable. One patient reported esophageal spasms and thoracic sensations at the time of the scheduled LES stimulations. This problem resolved after reduction of the stimulation amplitude to 2.5 mAmp. No further changes were made to the stimulation protocol at the follow-up.

## Discussion

This is the first one-year report of a prospective open-label study of electrical stimulation therapy of the lower esophageal sphincter in patients with GERD and ineffective esophageal motility. The results show that although patient satisfaction improved significantly, there was no reduction of acid exposure one year after surgery. Moreover, there were no adverse events or side effects such as dysphagia.

Health-related quality of life did significantly improve after surgery and the majority of patients remained satisfied at 12-month follow-up (composite GERD-HRQL improved from baseline 43 (22–47) to 12 (3.5–22.8). Taking into account, that follow-up pH metry did not show any reduction of acid exposure, this finding is of great interest. Several explanations could be possible.

First and foremost, there might be a placebo effect. Although this is less likely one year after surgery, questionnaires are typically of a subjective nature. Initially, the placebo effect was claimed for reducing symptoms of about 35% of the patients [15]. Currently, it is supposed that placebo effect occurs in almost every disease and goes far beyond the 35% published in 1955 [16, 17]. However, there are several studies concerning placebo effect in surgery [18]. Placebo effects are related to the patients’ expectation and the “meaning of surgery” [18]. This has to been differentiated from other non-specific effects such as the natural course of disease or unidentified parallel interventions [19]. Still, a placebo response to LES-EST cannot explain the entire divergence between HRQL and pH metry. But of course, other non-specific effects such as time effect or unknown parallel interventions may influence our findings.

Second, the GERD-HQRL score only represents the symptoms of regurgitation and heartburn. Those are the most frequent symptoms, but still, 52.9% of the patients were also reporting atypical symptoms like persistent cough, hoarseness, or burning in the mouth or throat. However, regarding heartburn and regurgitation, patient reported a significant improvement and the majority were satisfied with symptom control.

Third, electrical stimulation may influence the threshold of symptom perception. It was already stated that a possible explanation could be an interference with the afferent nerve transmission [11]. The combination of patient satisfaction and abnormal acid exposure endorses this theory. However, this possibility still remains to be evaluated.

Importantly, this is the first study, which fails to demonstrate normalization or even a significant improvement of acid exposure in the distal esophagus after LES-EST. In contrast to the two-year results of LES-EST published by Rodriguez et al., this unexpected finding asks for interpretation. The authors describe a significant improvement of acid exposure in the distal esophagus two years after LES-EST [11]. Still, they classify 61% of the patients as “abnormal distal esophageal pH.” As the patient cohort of Rodriguez did not include any patients with IEM, more advanced disease in our population may be responsible for that outcome. Recently, IEM was even associated with dysfunction of chemical clearance [20]. However, comparing our results to patients with defective esophageal motility after fundoplication at least some partial response in acid reduction was expected [21].

Regarding postoperative manometry, we did not see a significant improvement of the distal contractile integral. Nevertheless, we observed the return to regular esophageal motility in 3 patients after LES-EST. Some experimental studies show a possible recovery of esophageal motility after treatment of esophagitis [22, 23]. In a clinical study assessing motility before and after antireflux surgery, IEM resolved after fundoplication in 8.8% of the patients [24]. It is unclear if this is due to a direct effect of stimulation on the esophageal musculature or represents an indirect effect due to sufficient acid control. Follow-up manometry will help to elucidate this return to regular esophageal function more clearly.

Nonetheless, one main goal for antireflux surgery is patient satisfaction and its impact on quality of life. This consists of adequate symptom relief in combination with minimal side effects. While this patient group is at risk for postoperative dysphagia after antireflux surgery, we did not see any signs of negative side effects of LES-EST. This is interesting as it was shown that preoperative DCI is inversely correlated to postfundoplication dysphagia [25].

More than two thirds of our patients underwent additional hiatal hernia repair. As cruroplasty is known to provide significant antireflux effect itself, and subgroup analysis in our small group is not possible, our data are not able to discriminate between the antireflux effect of the LES-EST and the cruroplasty in the patients group that had received both [26].

Our study has some important limitations to address. First of all, the patient number permits only limited interpretation. Sample size calculation was done to detect improvement of GERD-HRQL. Still, an objective confirmation via EFT was to expect. Second, a possible placebo effect, especially in HRQL questionnaires, was already addressed and discussed. Concluding, three patients did not consent to EFT one year after surgery due to subjective improvement of GERD, which of course may influence the pH metry findings.

In summary, this study supports the concept that LES-EST can be especially offered to patients with esophageal dysmotility due to missing gastrointestinal side effects such as dysphagia. However, the study fails to demonstrate a decrease of esophageal acid exposure. Thus, we could not prove the effectiveness of LES-EST through the most objective and robust method, the esophageal pH metry. Furthermore, we failed to achieve improvement in distal LES contraction by LES-EST in this patient group. Patients with GERD and IEM remain a challenging patient group regarding surgical treatment. Further studies are needed to understand the mechanism of LES-EST and its role in GERD treatment.
